# Hsa_circ_0043278 functions as competitive endogenous RNA to enhance glioblastoma multiforme progression by sponging miR-638

**DOI:** 10.18632/aging.103603

**Published:** 2020-11-05

**Authors:** Zhi Wu, Maohua Zheng, Yonghong Zhang, Min Xie, Shilai Tian, Tao Ding, Lichao Li, Quanlin Guan

**Affiliations:** 1 Department of Neurosurgery, The First Hospital of Lanzhou University, Lanzhou 730000, China; 2 Department of Surgical Oncology, The First hospital of Lanzhou University, Lanzhou 730000, China

**Keywords:** glioblastoma multiforme, circ_0043278, Wnt/β-catenin signaling, migration, invasion

## Abstract

Circular RNAs have a critical function in the pathogenesis of many diseases and can function as competing endogenous RNA or miRNA sponges to inhibit miRNA and therefore upregulate the expression of target genes. However, little is known about the role of has_circRNA_0043278 (circ_0043278) in glioblastoma multiforme (GBM) and its potential downstream miRNA targets. This work validated that circ_0043278 is highly expressed in GMB cell lines and tissues, while knockdown circ_0043278 inhibited GBM cell migration, proliferation, and invasion *in* *vitro* and tumorigenesis *in* *vivo*. Dual-luciferase reporter assay determined that circ_0043278 directly sponged miR-638 to upregulate the expression of HOXA9, which can activate downstream Wnt/β-catenin signaling in GBM. Moreover, miR-638 inhibition reversed circ_0043278 silencing-induced impairment of malignant tumor behavior. These results showed that circ-0043278/miRNA-638/ Homeobox A9 (HOXA9) axis had a vital function in promoting GBM progression. Our findings may provide potential new targets for the diagnosis and therapy of GBM.

## INTRODUCTION

Glioma is a main class of primary brain tumor that leads to half of malignant brain tumor types [1]. The World Health Organization categorizes gliomas into four grades (from I to IV) according to overall survival (OS) and malignancy. Glioblastoma multiforme (GBM) is a malignant fast-growing grade IV glioma. GBM is the most typical adult malignant primary brain tumor that results in 60–80% of primary brain tumors [2], with a median OS of ~9–15 months and a five-year survival rate <10%, despite chemotherapy, radiotherapy, and surgical treatments [3–5]. Early prognosis and diagnosis are necessary for accurate GBM treatment. Due to poor outcomes after conventional treatments, it is necessary to discover novel therapeutic approaches and treatment policies for GBM, particularly those targeting complicated gene regulation networks.

Circular RNAs (circRNAs) belong to a new family of endogenous RNAs in mammalian cells. These include covalently closed continuous loops without 5ʹ end caps and 3ʹ end poly (A) tails [6, 7]. CircRNAs are mostly derived from exons, which are mainly found in the eukaryotic cell cytoplasm [8]. In addition, circRNA expression is disease-specific and organic [9]. CircRNAs have been formerly erroneously regarded as byproducts of splicing errors that were not associated with any specific mechanisms. Improvements in high-throughput sequencing technology led to investigations that determined that many human circRNAs might be involved in fetal development [10], myocardial infarction [11], and carcinomas [12]. CircRNAs have important functions in many diseases, such as tumors, by upregulating target gene expression via microRNA (miRNA) sponge action [13]. These characteristics make circRNAs potential candidates for tumor treatment and diagnosis, which are indispensable in medicine of tumor transformation. Previous research studies have indicated that circRNAs have the capability to inhibit or facilitate various tumor progression and development, including gliomas [14–16].

Many circRNAs have the potential to become diagnostic and prognostic biomarkers for some traits, especially in cancer [17–19]. CircRNA microarray data revealed that hsa_circRNA_0043278 (circ_0043278, gene symbol is TADA2A) is upregulated in pancreatic ductal adenocarcinoma [20]. CircTADA2A promotes osteosarcoma cell migration, proliferation, invasion, and metastasis by sponging miR-203a-3p [21].

The present study identified that circ_0043278 is upregulated in GBM cell lines and tissues. Knockdown circ_0043278 significantly inhibited cell migration, proliferation, and invasion. Study results verified that circ_0043278 upregulated Homeobox A9 (HOXA9) expression by absorbing miR-638 in GBM. This study provides a novel direction for clinical therapy of GBM.

## RESULTS

### Circ_0043278 is highly expressed in glioma cell lines and tissues while circ_0043278 downregulation inhibits glioma cell growth, migration and invasion

**Figure 1:**
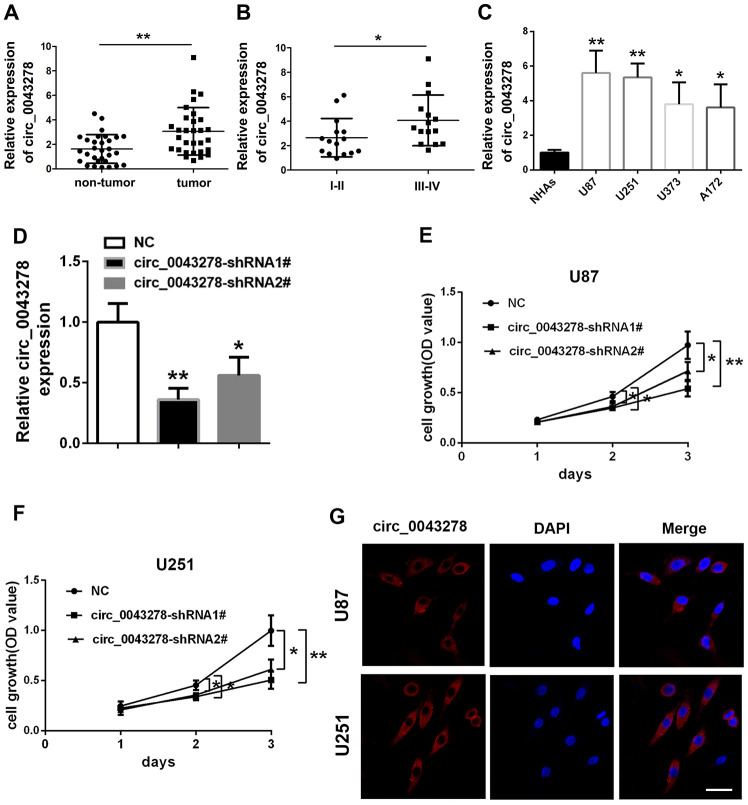
**Expression and verification of circ_0043278 in glioma cells and tissues.** qRT-PCR results for (**A**) circ_0043278 expression in glioma tumor tissues and their paired adjacent non-tumor tissues. Results represent the mean±SD for three independent experiments (N=30, **p<0.01, as determined by paired Student’s *t*-test); (**B**) circ_0043278 expression in glioma tissues of different grades. Results represent the mean±SD for three independent experiments (N=15 (I-II), N=15 (III-IV), *p<0.05, as determined by unpaired Student’s *t*-test); (**C**) circ_0043278 expression in four glioma cell lines and normal human astrocytes (NHAs). Results represent the mean±SD for three independent experiments (*p<0.05, **p<0.01, as determined by one-way ANOVA with Dunnett’s multiple comparisons test); (**D**) circ_0043278 expression after stable transfection of circ_0043278-shRNA1#/2#, or negative control (NC). Results represent the mean±SD for three independent experiments (*p<0.05, **p<0.01, as determined by one-way ANOVA with Dunnett’s multiple comparisons test); CCK-8 assay results demonstrated that (**E**, **F**) shRNA-mediated circ_0043278 silencing suppresses U87 and U251 cell growth. Results represent the mean±SD of three independent experiments (*p<0.05, **Pp<0.01, as determined by one-way ANOVA with Dunnett’s multiple comparison test); FISH results showed that (**G**) circ_0043278 was predominantly localized in the cytoplasm. Nuclei were stained with DAPI and circ_0043278 probes were labeled with Alexa Fluor 555 (scale bar = 50 μm in all panels).

**Figure 2:**
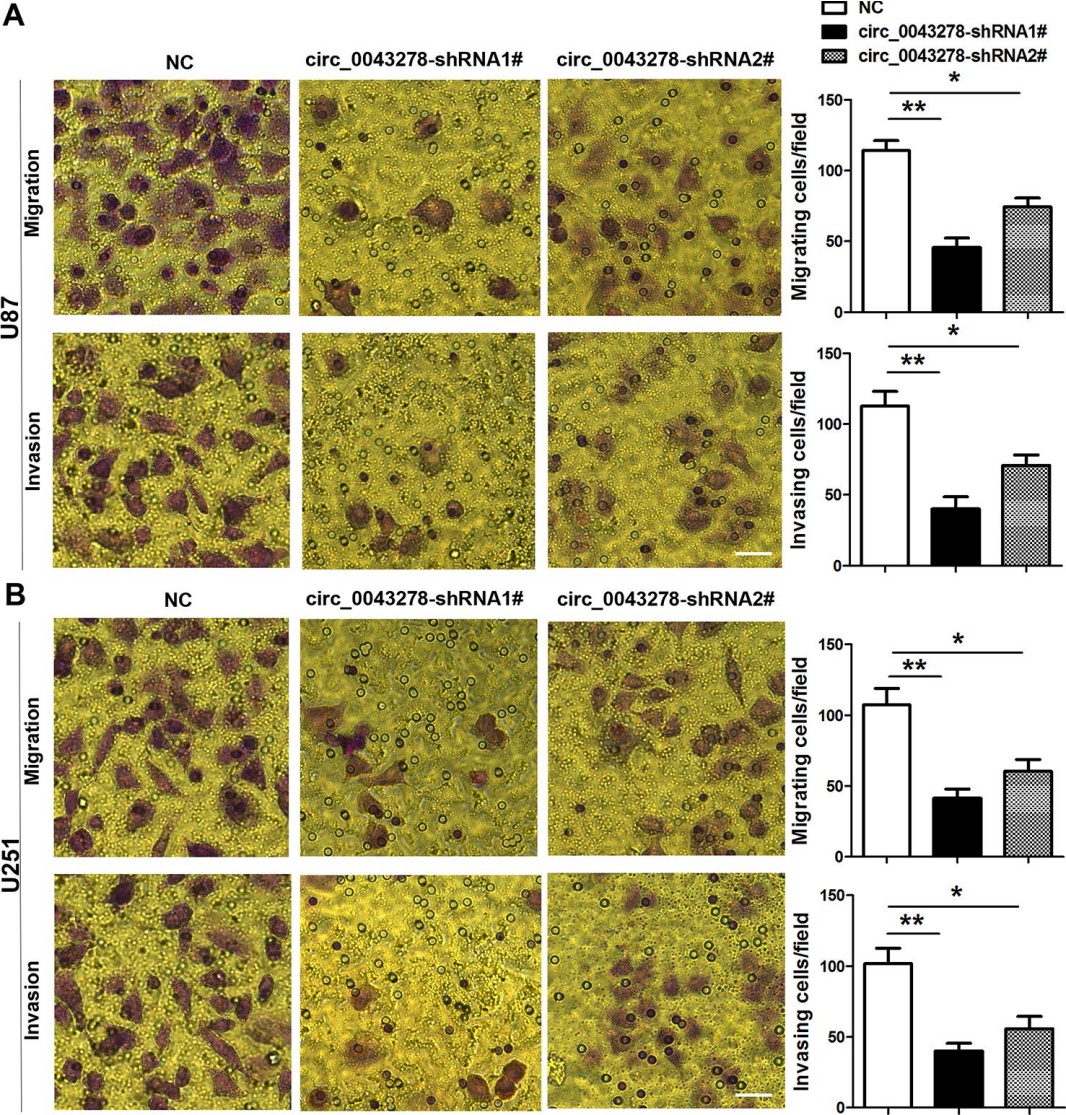
**Knockdown circ_0043278 inhibits glioma cell migration and invasion.** (**A**, **B**) Transwell assay results for U87 and U251 glioma cell invasion and migration. Results represent the mean±SD for three independent experiments (*p<0.05, **p<0.01, as determined by one-way ANOVA with Dunnett’s multiple comparisons test). NC, negative control. Scale bar = 50 μm in all panels.

The qRT-PCR analysis was performed in 30 pairs of human glioma tissues and their adjacent non-tumor tissue samples to confirm circ_0043278 expression in GBM. Circ_0043278 expression level was increased significantly in 30 glioma tissues compared to adjacent non-tumor tissues (t(29)=3.594, p=0.0012, Figure 1A). Moreover, circ_0043278 expression was higher in III–IV grade glioma tissues than in I–II grade tissues (t(28)=2.106, p=0.044, Figure 1B). Circ_0043278 expression was analyzed in human GBM cell lines. Circ_0043278 was higher in four glioma cell lines (A172, U251, U87, and U373) than in normal human astrocytes (NHAs) (F(4,10)=8.776, p=0.003). Among the four glioma cell lines, U87 and U251 cells exhibited the highest circ_0043278 levels (Figure 1C). Two circ_0043278 small hairpin RNA (shRNA1# or 2#) products were designed to specifically target circRNA junction sites, while negative control (NC) products were used as a control. They were then transfected into U87 cells and assessed using qRT-PCR. Circ_0043278 expression was significantly silenced by shRNAs (F(2,6)=17.51, p=0.003), while circ_0043278-shRNA1# had the best knockdown efficiency (Figure 1D). CCK-8 assay determined that silencing circ_0043278 inhibited cell proliferation both in U87 and U251 cells after 24 h (F(2,6)=0.642, p=0.559; F(2,6)=4.995, p=0.053; respectively), 48 h (F(2,6)=8.564, p=0.018; F(2,6)=8.471, p=0.018; respectively), and 72 h (F(2,6)=14.90, p=0.005; F(2,6)=16.16, p=0.004; respectively; Figure 1E and 1F). Furthermore, RNA fluorescence in situ hybridization (FISH) suggested that circ_0043278 was mainly localized in the U87 and U251 cell cytoplasm (Figure 1G). Additionally, transwell assays showed that circ_0043278 knockdown significantly reduced both U87 cell migration (F(2,6)=22.04, p=0.002)and invasion (F(2,6)=16.57, p=0.004) and U251 cell migration (F(2,6)=13.89, p=0.0026) and invasion (F(2,6)=13.50, p=0.006; Figure 2A and 2B). These results demonstrated that knockdown circ_0043278 inhibits the proliferation, migration, and invasion of GBM cells.

### MiR-638 is downregulated in glioma tissues while miR-638 overexpression promotes glioma cell growth, migration and invasion

**Figure 3:**
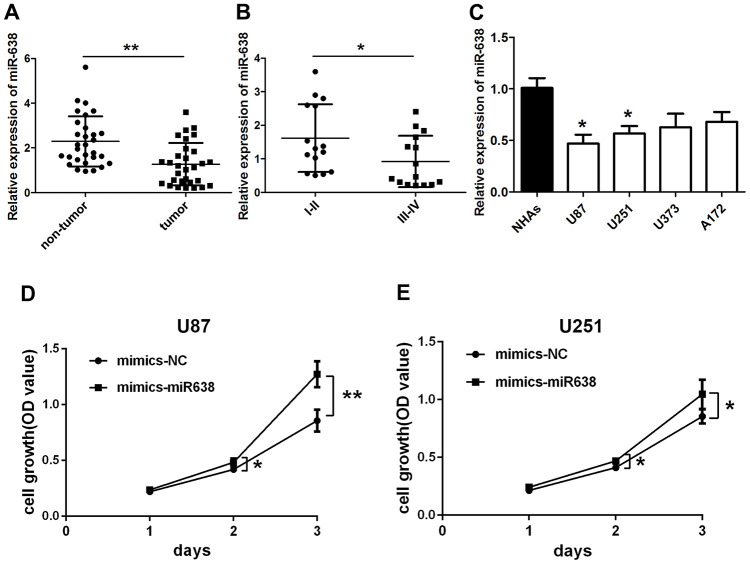
**Overexpression of miR-638 promotes glioma cell growth.** qRT-PCR results for (**A**) miR-638 expression in glioma tumor tissues and their paired adjacent non-tumor tissues. Results represent the mean±SD for three independent experiments (N=30, **p<0.01, as determined by paired Student’s *t*-test); (**B**) miR-638 expression in glioma tissues of different grades. Results represent the mean±SD for three independent experiments (N=15 (I-II), N=15 (III-IV), *p<0.05, as determined by unpaired Student’s *t*-test); (**C**) miR-638 expression in four glioma cell lines and NHAs cells. Results represent the mean±SD for three independent experiments (*p<0.05, as determined by one-way ANOVA with Dunnett’s multiple comparisons test); (**D**, **E**) U87 and U251 cells were transfected with miR-638 mimics. Cell growth was determined using CCK-8 assay. Results represent the mean±SD for three independent experiments (*p<0.05, **p<0.01, as determined by unpaired Student’s *t*-test).

**Figure 4:**
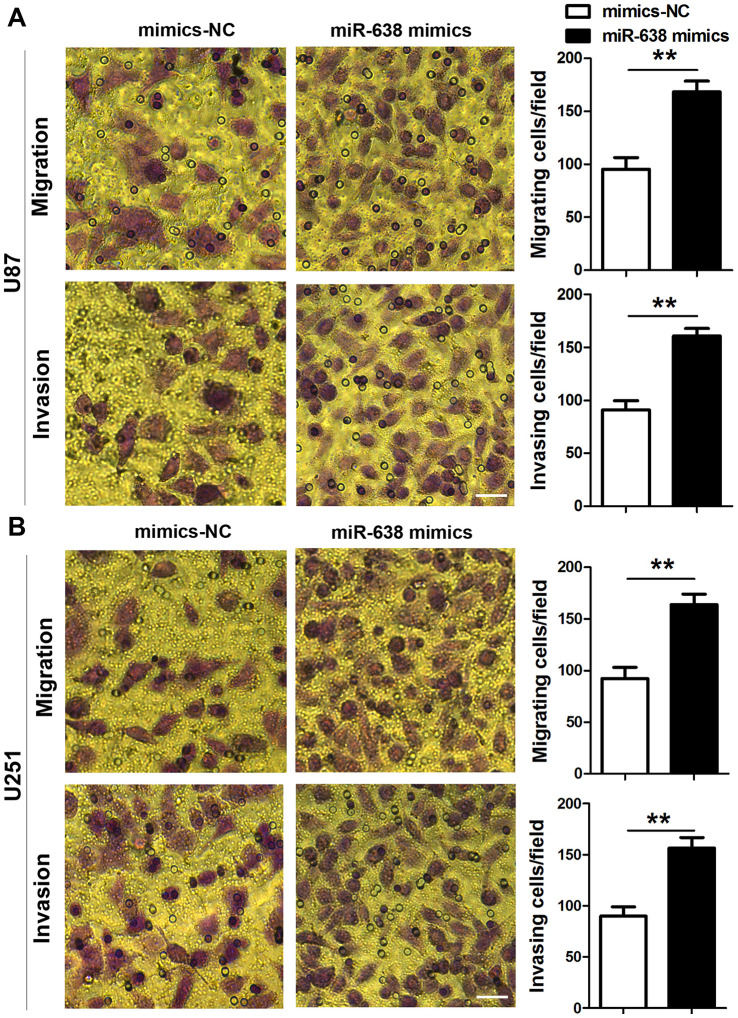
**Overexpression of miR-638 promotes glioma cell migration and invasion.** (**A**, **B**) U87 and U251 glioma cells were transfected with miR-638 mimics, and both cells migration and invasion were determined using transwell assays. Results represent the mean±SD for three independent experiments (**p<0.01; as determined by unpaired Student’s *t*-test). Scale bar = 50 μm in all panels.

Zheng et al. reported that miR-638 expression is down-regulated in glioma tissues compared to normal tissues [22]. The current study also revealed a significantly lower miR-638 expression in glioma tissues compared to adjacent non-tumor tissues (t(29)=3.595, p=0.0012, Figure 3A). Moreover, this expression was lower in high-grade than in low-grade glioma tissues (t(28)=2.127, p=0.042, Figure 3B). Unlike circ_0043278, miR-638 expression levels in U87, A172, U251, and U373 cells were significantly lower than those in NHAs (F(4,10)=4.330, p=0.027, Figure 3C). Therefore, miR-638 mimics were used to overexpress miR-638 and transfected into both U87 and U251 cells, where cell growth was analyzed using a CCK-8 assay. The results indicated that overexpression of miR-638 increased both U87 and U251 cell proliferation (Figure 3D and 3E) after 24 h (t(4)=1.269, p=0.273; t(4)=1.181, p=0.303; respectively), 48 h (t(4)=2.853, p=0.046; t(4)=3.229, p=0.032; respectively) and 72 h (t(4)=4.754, p=0.009; t(4)=2.797, p=0.049; respectively). Furthermore, transwell assays showed that overexpression miR-638 increased both U87 and U251 cell migration (t(4)=4.912, p=0.008; t(4)=4.894, p=0.008; respectively; Figure 4A) and invasion (t(4)=6.261, p=0.003; t(4)=4.927, p=0.008; respectively; Figure 4B). These results demonstrated that miR-638 overexpression increases the GBM cells proliferation, migration, and invasion.

### Circ_0043278 targets on miR-638

**Figure 5:**
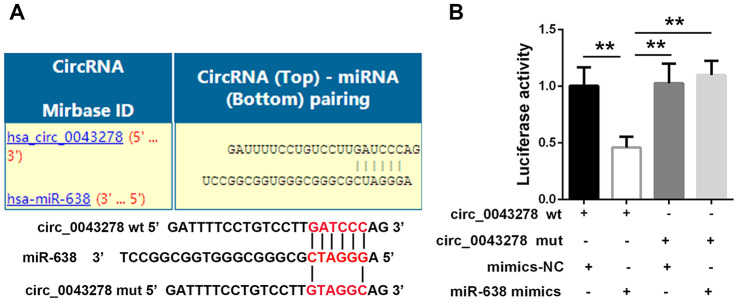
**Circ_0043278 targets on miR-638.** (**A**) Putative circ_0043278 binding sites in miR-638; (**B**) Luciferase reporter plasmid containing wt or mut circ_0043278 was co-transfected into HEK-293 cells with miR-638 or NC mimics. Luciferase activity was determined 24 h after transfection via dual-luciferase assay and normalized to Renilla luciferase activity. Results represent the mean±SD for three independent experiments (**p<0.01, as determined by one-way ANOVA with Bonferroni’s multiple comparisons test).

Science that circ-0043278 is predominantly distributed in the cytoplasmic fraction, it was hypothesized that circ-0043278 may act as a competitive endogenous RNA (ceRNA) in biological processes. Based on the online predictions of circ-interactome (https://circinteractome.nia.nih.gov) and analysis using the circ-interactome database, circ_0043278 shares putative binding sites with miR-638 (Figure 5A). To explore a potential interaction between circ-0043278 and miRNA-638, dual-luciferase reporter plasmids carrying a fragment of the mutant (mut) or wild-type (wt) circ-0043278 sequence and the predicted miR-638 recognition site were constructed. Luciferase activity was markedly decreased in 293 cells co-transfected with circ-0043278-wt and miR-638 mimics. However, no significant differences in luciferase activity were present in cells co-transfected with circ-0043278-mut and miR-638 mimics (F(3,8)=12.84, p=0.002, Figure 5B). These results suggest that circ_0043278 sponges miR-638.

### MiR-638 inhibitor reverses cell migration and invasion induced by circ_0043278 silencing

**Figure 6:**
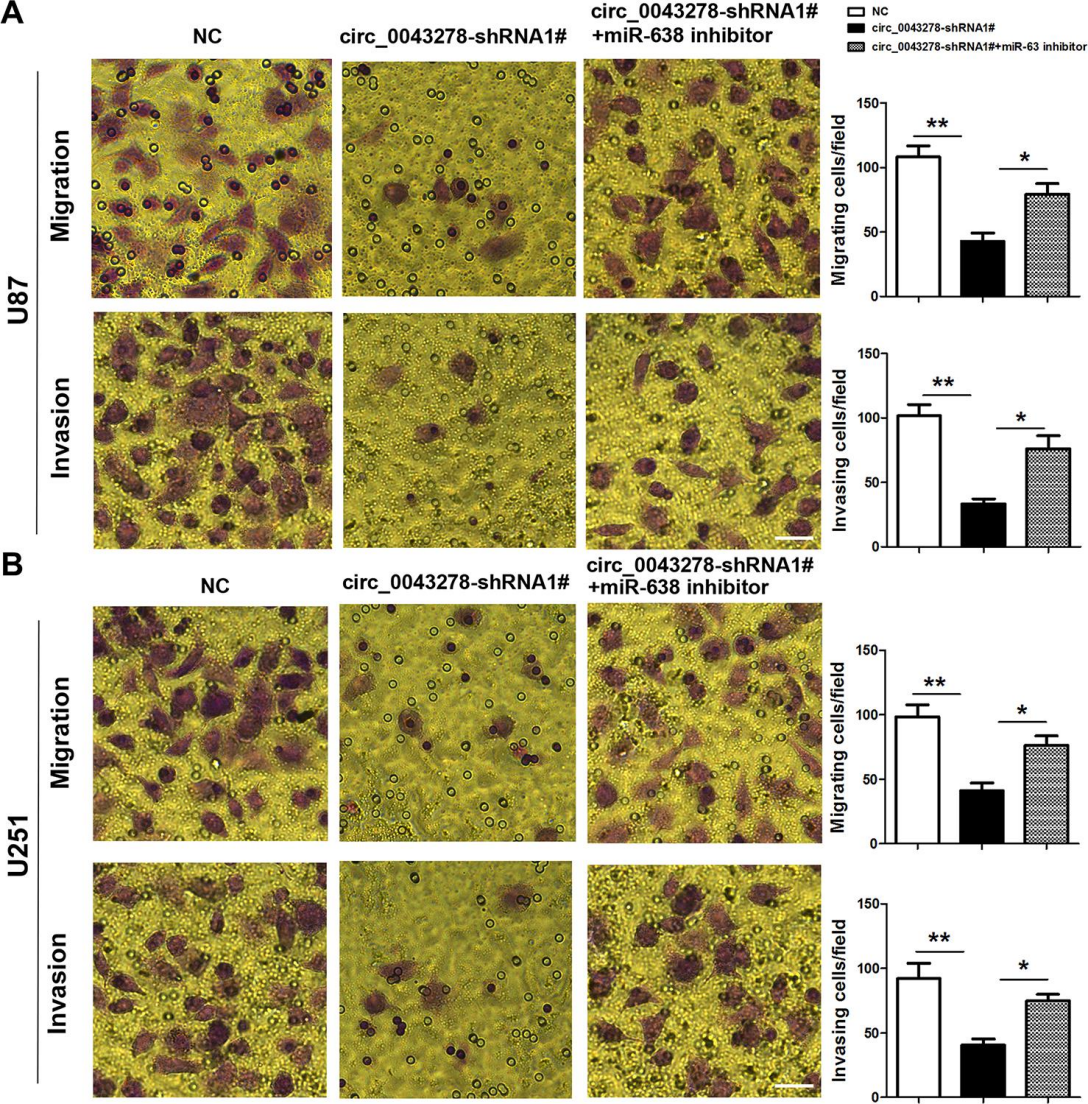
**MiR-638 inhibitor reverses circ_0043278-shRNA1#-induced glioma cell invasion and migration attenuation.** (**A**, **B**) U87 and U251 cells were stably transfected with NC or cic_0043278-shRNA1# or co-transfected with cic_0043278-shRNA1# and miR-638 inhibitor. Migration and invasion were evaluated using transwell assays. Results represent the mean±SD for three independent experiments (*p<0.05, **p<0.01, as determined by one-way ANOVA with Bonferroni’s multiple comparisons test). Scale bar = 50 μm in all panels.

Rescue experiments were performed to detect whether circ_0043278 enhances migration and invasion in gliomas by interacting with miR-638. U87 and U251 cells were stably transfected with circ_004327-shRNA1# or co-transfected with circ_004327-shRNA1# and miR-638 inhibitor. Cell migration and invasion were evaluated using transwell assays (Figure 6A and 6B). The results indicated that circ_0043278 knockdown inhibits glioma cell migration and invasion, while exogenous miR-638 expression downregulation blocks both U87 and U251 cell migration (F(2,6)=17.37, p=0.003; F(2,6)=13.71, p=0.006; respectively) and invasion (F(2,6)=18.28, p=0.003; F(2,6)=10.70, p=0.011; respectively) impairments.

### Circ-0043278 functions as ceRNA to sponge miR-638 to regulate HOXA9 expression

**Figure 7:**
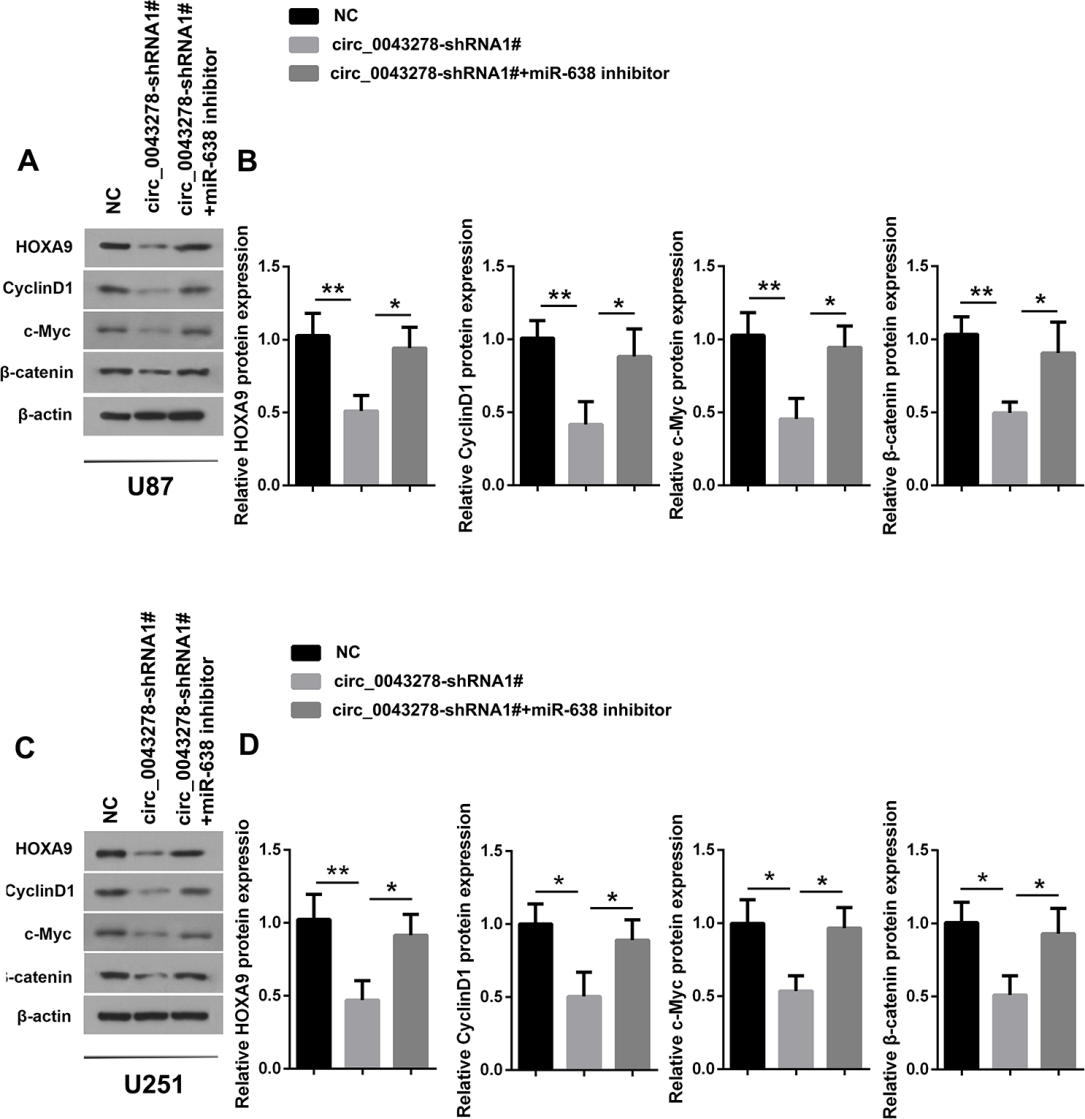
**Circ_0043278/MiR-638 regulates HOXA9/Wnt/β-catenin signaling pathway expression.** (**A**–**D**) U87 and U251 cells were stably transfected with NC or cic_0043278-shRNA1# or co-transfected with cic_0043278-shRNA1# and miR-638 inhibitor. HOXA9 and Wnt/β-catenin signaling protein expression were evaluated using western blotting. Results represent the mean±SD for three independent experiments (*p<0.05, **p<0.01, as determined by one-way ANOVA with Dunnett’s multiple comparisons test).

It has been shown that HOXA genes are aberrantly activated within confined chromosomal domains in GBM, and that HOXA9 expression is associated with poorer survival and is controlled by PI3K mediated transcriptional process [23]. Zhang et al. reported that HOXA9 can act downstream Wnt/β-catenin signaling and promote cell proliferation in human osteosarcoma [24]. It has also been reported that miR-638 targets HOXA9 3’UTR directly, downregulates HOXA9 expression, and thus inhibits the two Wnt signaling effectors c-Myc and Cyclin D1, while overexpression of HOXA9 can partially abolish the miR-638 effects in glioma [22]. The present study determined that circ_0043278 silencing inhibits HOXA9 protein expression in both U87 and U251 cells (F(2,6)=12.43, p=0.0073; F(2,6)=11.52, p=0.0088; respectively), as well as c-Myc (F(2,6)=13.12, p=0.0064; F(2,6)=10.67, p=0.0106; respectively), Cyclin D1 (F(2,6)=11.84, p=0.0083; F(2,6)=9.392, p=0.0142; respectively) and β-catenin protein (F(2,6)=11.09, p=0.0097; F(2,6)=9.609, p=0.0135; respectively), which are Wnt/β-catenin pathway target genes. Furthermore, miR-638 inhibitor can partially rescue the effects of circ_0043278 silencing (Figure 7A and 7D). In summary, these results suggest that circ_0043278 acts as a ceRNA to sponge miR-638 to upregulate HOXA9, thus activating the Wnt/β-catenin signaling pathway.

### Circ_0043278 silencing inhibits tumor growth *in vivo*

**Figure 8:**
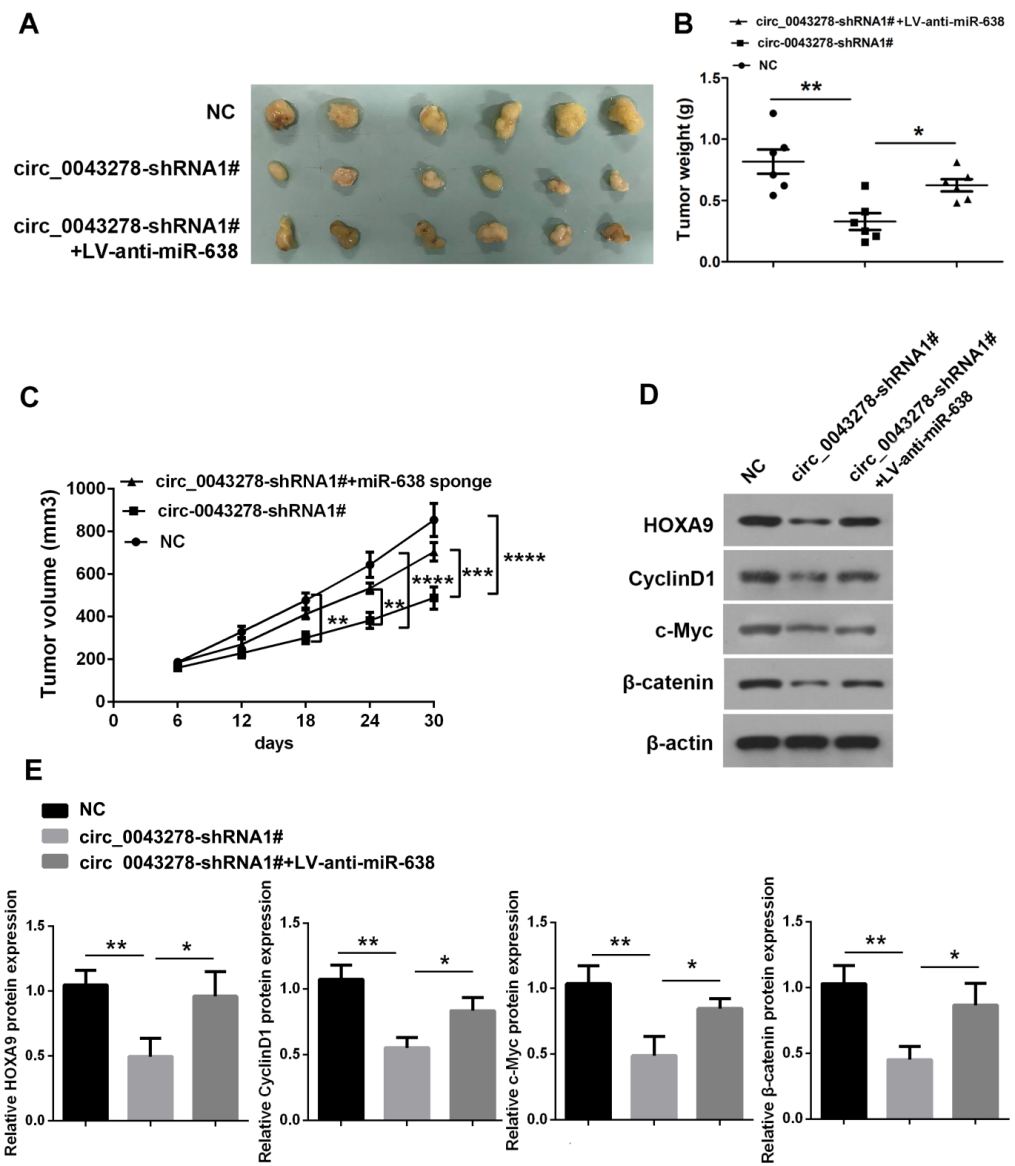
**Circ_0043278 acts as miR-638 sponge to enhance tumorigenesis *invivo*.** (**A**) Images of mouse tumors one month after implantation with U87 cells stably transfected with NC or cic_0043278-shRNA1# or co-transfected with cic_0043278-shRNA1# and LV-anti-miR-638 subcutaneously. Tumors were dissected and photographed after one month; (**B**) Tumor weight was calculated after euthanasia (N = 6 in each group, *p<0.05, **p<0.01, as determined by one-way ANOVA with Bonferroni’s multiple comparisons test); (**C**) Tumor volumes were recorded every six days after mice were injected with stable glioma cells (N = 6 in each group, **p<0.01, ***p<0.001, *** p<0.0001, as determined by two-way ANOVA with Dunnett’s multiple comparisons test); (**D**, **E**) Western blotting was used to detect HOXA9 protein levels and Wnt/β-catenin signaling. Results represent the mean±SD for three independent experiments (*p<0.05, **p<0.01, as determined by one-way ANOVA with Dunnett’s multiple comparisons test).

To investigate the functions of circ_0043278 and miR-638 in *vivo*, a xenograft tumor model was established. We used stable U87 cells transfected with NC or circ_0043278-shRNA1# or co-transfected with LV-anti-miR-638. The tumors derived from the circ_0043278 silencing cells were much smaller in size (Figure 8A), weighed less than the control tumors (F(2,15)=10.59, p=0.001, Figure 8B) and had tumor volumes (F(2,15)=9.811, p=0.0019, Figure 8C). The miR-638 sponge significantly reversed the impairments in tumor size and weight caused by circ_0043278 knockdown. Moreover, circ_0043278 silencing predominantly reduced protein expression of HOXA9 (F(2,6)=11.45, p=0.0089), as well as c-Myc (F(2,6)=15.68, p=0.0041), Cyclin D1 (F(2,6)=21.46, p=0.0018) and β-catenin protein (F(2,6)=14.36, p=0.0052), which could be reversed by the miR-638 sponge (Figure 8D and 8E).

## DISCUSSION

CircRNAs belong to a class of single-stranded RNA molecules with a covalently closed loop structure. They have been characterized by high stability, abundance, conservation, and display tissue stage-specific expression. Furthermore, based on the abundance in distinct body fluids or exosomes, circRNAs present novel biomarkers and targets for the diagnosis and prognosis of cancers [25]. CircRNAs have indispensable functions in different biological processes, especially carcinogenesis, cancer metastasis, and progression in lung adenocarcinoma [26], hepatocellular carcinoma [27], and gastric cancer [28]. Recently, several circRNAs have been reported to play important roles in human glioma. CircRNAs such as circSMARCA5 and circCFH, are expressed in a glioma- specific pattern. These circRNAs may be used as tumor biomarkers. CircNFIX and circNT5E served as tumor promoter in glioma, while circFBXW7 and circSHPRH were reported to function as tumor suppressors [29]. Wu et al. found that circTADA2A, also named circ_0043278, target miR-203 to upregulate CREB3 expression, thus leading to cell migration, invasion, and proliferation in osteosarcoma [21]. Zhang et al. used circRNA microarray dataset analysis and found that circ_0043278 was upregulated in pancreatic ductal adenocarcinoma [20]. Nevertheless, circ_0043278 functions in GBM remain unknown. The current study focused on the role of circ_0043278 in GBM mechanisms of cell proliferation, invasion, and migration. Compared to normal human astrocytes, circ-0043278 is highly expressed in the GBM cell lines. Knockdown of circ-0043278 markedly inhibited cell proliferation, migration, and invasion *in* *vitro* and tumorigenesis *in* *vivo*, indicating that circ_0043278 is a positive tumor promoter in GBM.

As a newly discovered type of noncoding RNA, circRNAs share many functions with traditional noncoding RNAs. Recently, circRNAs have been reported to function as miRNA sponges, protein sponges, coding RNAs or scaffolds for protein complexes [29]. Long non-coding RNAs (lncRNAs) function as ceRNA to upregulate gene expression by targeting miRNAs in GBM [30]. CircRNAs also can function asceRNAsor miRNA sponges to inhibit miRNA and therefore upregulate the expression of target genes via miRNA response elements [31]. MiRNAs are small non-coding RNAs that are 18–24 nucleotides long and function during gene expression regulation [32]. Translation of targeted mRNAs is inhibited post-transcriptionally via miRNA binding to 3'UTR sequences [33, 34]. For example, circRNA-5692 binds to miR-328-5p to enhance DAB2IP expression and thus inhibits cell migration, proliferation, and invasion in hepatocellular carcinoma [35]. Circ_0000285 acts as a ceRNA that increases TGFB2 expression by sponging hsa-miRNA-599 in osteosarcoma [36]. In the current study, miR-638 was downregulated in glioma cells and tissues, while a luciferase reporter assay indicated that circ_0043278 directly targets miR-638.

In addition, HOXA9 was reported to activate the Wnt/β-catenin signaling pathway and is directly targeted by miR-638 [24]. The Wnt/β-catenin pathway is a highly conserved signaling cascade that takes part in different cellular processes, such as cell differentiation, proliferation, adhesion, survival, and migration [37].

Wnt/β-catenin signaling has been recently found to be related to EMT-like states [38], cancer stem cell biology [39], self-renewal [40], and chemo-resistance [41] in some cancers. Sun et al. reported that some circRNAs affect the proliferation and invasion of gliomas through cancer-associated signaling pathways. For example, circ-0000177 activates the wnt/β-catenin pathway through miR-638/FZD7, while circTTBK2, circSHKBP1 and circHIPK3 activate PI3K/AKT and MARK/ERK signaling pathway through miR217/HNF1β, miR544a/ FOXP1, and miR379/FOXP2, respectively [25]. The present study found that circ_0043278 functions as a ceRNA that increases HOXA9 expression and Wnt/β-catenin signaling pathway by sponging miR-638.

In conclusion, this study demonstrated that circ_ 0043278 functions as a ceRNA to enhance GBM progression by sponging miR-638. The results indicated that circ_0043278 may function as a new biomarker, which provides a novel direction for GBM clinical diagnosis and treatment.

## MATERIALS AND METHODS

### Patient samples

A total of 30 patients were enrolled in the study. The patients had surgeries to resect glioma tumors, as well as adjacent non-tumor tissues at the First Hospital of Lanzhou University between September 2017 and December 2018. Tumor specimens were snap frozen in liquid nitrogen and kept at -80°C until further use. Tissue sections were inspected by three pathologists during diagnosis to identify tissue variants. Ethics Committee at the First Hospital of Lanzhou University approved the study. Consent forms were obtained from all patients.

### Cell culture

Human U87, U251, A172, and U373 glioblastoma cell lines were purchased from the ATCC (Manassas, VA, USA). Primary normal human astrocytes (NHAs) were from Sciencell Research Laboratories (Carlsbad, CA, USA). NHAs were cultured in Dulbecco’s modified Eagle’s medium (DMEM), while all cultured glioma cell lines were cultured in DMEM-F12. Both mediums were supplemented with 10% fetal bovine serum (FBS, Gibco, Grand Island, NY, USA) and incubated at 37°C and 5% CO_2_. No mycoplasma contamination was detected in all cell lines by the MycAwayTM Plus-Color One-Step Mycoplasma Detection Kit (Yeasen, Shanghai, China).

### Cell transfection

Two scrambled miRNAs were used as negative controls. Mimic negative control (NC) was used for mimic miR-638, while NC inhibitor was used for miR-638 inhibitor. MiR-638 mimic was used for miR-638 overexpression, while miR-638 inhibitor was used for miR-638 knockdown, which were purchased from Ribobio (Guangzhou, China). Cells were transfected with 100 nM miR-638 mimics and inhibitors using Lipofectamine RNAiMAX reagent (Invitrogen, Carlsbad, CA, USA). To knockdown circ_0043278, small hairpin RNAs (shRNAs) targeting circ_0043278 lentivirus (circ_0043278-shRNA1#/2#) and a miR-638 inhibitor lentivirus (LV-anti-miR-638) were obtained from GeneChem (Shanghai, China). Lentivirus was validated, concentrated, and ultracentrifuged prior to addition to cell culture medium. After infection, cells were treated with puromycin (Gibco, Grand Island, USA) for one week to select for stably transfected cells.

### RNase R treatment, RNA extraction, and quantitative real-time PCR (qRT-PCR)

Total RNA samples were extracted from tissues and cells using TRIzol (Invitrogen, Carlsbad, USA) following standard procedures. For the RNase R treatment, 2 mg of total RNA were incubated for fifteen minutes at 37°C with or without 3 U/mg RNase R (Epicentre Technologies, Madison, USA). SYBR Premix Ex Taq II (TaKaRa) and script RT reagent kit (TaKaRa, Dalian, China) were used for the mRNA and circRNA analyses. Reactions were measured using a Roche LightCycler® 480II PCR instrument (Basel, Switzerland) following standard procedures. β-actin was used as a standard internal control. For the miRNA analyses, samples were treated with DNase I to remove genomic DNA, while cDNA was synthesized by leveraging the Mir-X miR First-Strand synthesis kit (TaKaRa). SYBR Premix Ex Taq II (TaKaRa) was used for qRT-PCR. U6 was employed as an internal standard control. Relative RNA expression levels were analyzed using a 2^-ΔΔCt^ method as previously described [21].

### Western blot (WB) analysis

Cells were lysed in the RIPA lysis buffer that included a protease inhibitor cocktail on ice. An equivalent amount of total protein was separated from various samples using SDS-PAGE gels at 100 V for 1.5 h and then transferred onto 0.22-μm polyvinylidene difluoride membranes at 280 mA for 1.5 h. The membranes were blocked with 5% non-fat milk for 1 h and then incubated overnight at 4°C with primary antibodies. The primary antibodies were: anti-HOXA9 (ab191178, 1:500), anti-CyclinD1 (ab134175, 1:5000), anti-c-Myc (ab185656, 1:1000), and anti-β-catenin (ab16051, 1:1000; all from Abcam, San Francisco, USA). Membranes were washed and then treated with goat anti-rabbit IgG conjugated with horseradish peroxidase (HRP) for 1 h at 37°C. Protein bands were visualized using chemiluminescence HRP substrate, where β-actin served as an internal control. Blot density was then quantified using Image J software.

### Fluorescence in situ hybridization (FISH)

Alexa Fluor 555-labeled circ_0043278 probes were designed and synthesized by leveraging RiboBio (Guangzhou, China). Probe signals were determined using a FISH kit (RiboBio, Guangzhou, China) following previously established guidelines [21]. Images were acquired using a fluorescence microscope (×200, Olympus, Dojindo, Japan).

### Cell proliferation assay

Cell proliferation was determined using a cell counting kit-8 (CCK-8; Dojindo, Japan). A total of 2 × 10^3^ cells/well were seeded into 96-well plates for overnight culture. Afterwards, 10 μL of the CCK-8 reagent were added for cell proliferation quantification at A450 nm after 24, 48, or 72 h using an Epoch Microplate spectrophotometer (BioTek, Winooski, USA).

### Cell migration and invasion assays

Migration and invasion capabilities were determined using a transwell assay (Corning, USA). For cell migration, culture inserts with 8.0-μM pores (Transwell, NY, USA) were placed into 24-well culture plates. Cell invasion assay was performed using a 40 μl 1:4 mixture of Matrigel Basement Membrane Matrix (Corning) according to the manufacturer’s protocols. Briefly, 200 μL of serum-free DMEM-F12 medium containing 5×104 treated cells for the migration assay, or 1 ×105 cells/well for the invasion assay were added to the upper chamber. Then, 600 μL of DMEM-F12 containing 10% FBS were added into the lower chamber. After incubation in a humidified atmosphere for 24 h, non-migrated or non-invaded cells were removed by scraping the upper surface of the membranes with a cotton swab, cells attached to the lower membrane were fixed in 4% paraformaldehyde at room temperature for 10 min and stained with 0.1% crystal violet at room temperature for 20 min. Five random fields of view were evaluated under a microscope with magnification of ×200.

### Luciferase reporter assay

Wild-type (wt) or mutant (mut) seed circ_0043278 regions were designed to include putative target sites for miR-638 (Shanghai GeneChem, Shanghai, China) and cloned into the pGL3 vector. HEK-293 cells were co-transfected with the reporter plasmid and miR-638 mimics or mimics NC (RiboBio). After transfection for two days, luciferase activity was analyzed with a Dual Luciferase Reporter Assay System (Promega) and normalized using Renilla luciferase activity.

### Subcutaneous tumor models

Animal Ethics Committee at the First Hospital of Lanzhou University approved all animal experiments. All protocols were established following provided guidelines. Five-week-old male nude mice were purchased from Shanghai SLAC Laboratory Animal Co. Ltd. (Shanghai, China) and housed in laminar airflow cabinets under pathogen-free conditions. Animals were randomly divided into three groups (NC, circ_0043278-shRNA1# and circ_0043278-shRNA1#+LV-anti-miR-638) (six in each group). Tumor formation was initiated via subcutaneous injection of infected U87 cells (2× 10^6^ cells) into nude mouse flanks. Tumor volume was computer using an equation, where volume = length × width^2^/2. Tumor volume was measured every six days. After 30 days, mice were euthanized and tumor grafts were excised, weighed, and harvested. Proteins were extracted from the one-half of tumors, and the expression of HOXA9, CyclinD1, c-Myc and β-catenin were measured by WB. The other half was fixed in 4% paraformaldehyde.

### Statistical analysis

Results are presented as the mean ± standard deviation (SD) from three independent experiments performed in triplicate (n=3). Statistical analysis was performed using GraphPad Prism 6 (GraphPad software, USA). Student’s t-test (paired or unpaired) was used to compare differences between two groups and one-way or two- way analysis of variance (ANOVA) was used to evaluate differences among more than two groups. p values<0.05 were considered statistically significant.
